# Double Hormone Syndromes in Two Pancreatic Neuroendocrine Tumours

**DOI:** 10.1155/1996/70392

**Published:** 1996

**Authors:** J. G. Geoghegan, R. Howerton, W. A. Ratcliffe, R. C. N. Wllliamson

**Affiliations:** 1 Department of Surgery Royal Postgraduate Medical School Hammersmith Hospital Du Cane Road London Wl20NN United Kingdom; 2 Wolfson Research Laboratory Queen Elizabeth Medical Centre Birmingham B15 2TH United Kingdom


*HPB Surgery*, 1996, Vol.9, pp.261–263
Reprints available directly from the publisher
Photocopying permitted by license only

© 1996 OPA (Overseas Publishers Association)
Amsterdam B.V. Published in The Netherlands
by Harwood Academic Publishers GmbH
Printed in Malaysia

### CASE REPORT

### Double Hormone Syndromes in Two Pancreatic Neuroendocrine Tumours

J. G. GEOGHEGAN, R. HOWERTON, W. A. RATCLIFFE* and R. C. N. WILLIAMSON

Department of Surgery, Royal Postgraduate Medical School, Hammersmith Hospital, Du Cane Road, London W12 ONN
*Wolfson Research Laboratory, Queen Elizabeth Medical Centre, Birmingham B15 2TH

*(Received 28 October 1993)*

### INTRODUCTION

Neuroendocrine tumours of the pancreas have an annual incidence of approximately 1 per 100,000 population. Up to 85% of these tumours secrete peptides, many of which cause characteristic clinical syndromes. While pancreatic neuroendocrine tumours frequently express immunoreactivity for multiple peptides, they rarely produce more than one clinical syndrome due to excess peptide secretion. We report two patients with pancreatic neuroendocrine neoplasia in each of whom there were characteristic clinical features due to hypersecretion of two bioactive peptides.

### CASE NO: 1

A 41-year-old man with recently diagnosed sarcoidosis underwent cholecystectomy for gallstones in 1986. At operation he was found to have multiple metastatic lesions throughout the liver with no identifiable primary site; histological examination showed neuroendocrine tumour. A gut hormone screen at that time was normal, but postoperative CT scan demonstrated a lesion in the tail of pancreas. Thereafter he attended a homeopathy clinic and declined conventional medical treatment.

*Correspondence to*: Professor R.C.N. Williamson, Department of Surgery, Royal Postgraduate Medical School, Hammersmith Hospital, Du Cane Road, London W12 ONN, U.K.

In July 1990 he developed symptomatic hypercalcaemia (4.5 mmol/l, normal 2.1–2.6 mmol/l), which was treated with forced diuresis, calcitonin and biphosphonates. Serum parathyroid hormone was undetectable, but parathyroid hormone-related protein (PTHRP) was elevated at 7.8 pmol/l (normal < 0.5 pmol/l). Somatostatin was also markedly elevated at 774 pmol/l (normal < 100 pmol/l); vasoactive intestinal peptide, pancreatic polypeptide, gastrin, glucagon, and neurotensin levels were all normal. Hepatic artery embolisation reduced the PTHRP level to 6.1 pmol/l, and at a second embolisation the splenic artery and an accessory artery to the left liver from the left gastric artery were occluded; thereafter serum calcium returned to normal. In December 1990 he was noted to be mildly diabetic and was started on glibenclamide.

In October 1991 the patient returned with severe hypercalcaemia (4.1 mmol/l) and was transferred to this unit for surgical debulking of his tumour. At operation a 20 cm tumour was found arising from the body and tail of the pancreas involving the left kidney and splenic flexure of colon. An *en bloc* distal pancreatectomy, splenectomy, left nephrectomy and left hemicolectomy was performed, from which he made an uncomplicated recovery. By the seventh postoperative day the serum calcium was 2.7 mmol/l and PTHRP was 1.1 pmol/l; however somatostatin remained elevated at 1141 pmol/l. Histological examination of tumour tissue showed typical features of a pancreatic neuroendocrine tumour with nests of polygonal cells and a focal trabecular pattern. Vascular invasion and lymph node involvement were noted. Immunohistochemical staining of the resected tumour was positive for neuron specific enolase, chromogranin and somatostatin and was negative for pancreatic polypeptide, insulin, and VIP.

In February 1992 he was readmitted to hospital with severe hypercalcaemia associated with seizures. His general condition deteriorated and he died within three weeks.

### CASE NO: 2

A 68-year-old man presented with a long history of recurrent peptic ulceration. He had undergone truncal vagotomy and pyloroplasty in 1982, antrectomy in 1983 and subtotal gastrectomy in 1986. In 1987 his gastrin level was 257 pmol/l (normal < 40 pmol/l) indicating that he had Zollinger-Ellison syndrome. Ultrasound and CT scanning failed to localise a gastrinoma, and his symptoms were controlled with omeprazole. In April 1991, he began to experience symptoms of severe hypoglycaemia. A supervised fast in hospital precipitated hypoglycaemia with inappropriate hyperinsulinism (glucose < 2.0 mmol/l; insulin 119 mU/l, reference range 3-20 mU/l; C-peptide 1214 pmol/l). Serum gastrin was 308 pmol/l. CT scan now showed a 14 × 18 cm tumour in the tail of pancreas with at least one liver metastasis, and visceral angiography confirmed multiple hepatic metastases. Distal pancreatectomy and splenectomy was performed together with enucleation of four liver metastases, thereby removing all macroscopic disease. Postoperative gastrin level was 4 pmol/l. During a 24 hour fast the blood glucose remained above 5.6 mmol/l. Histological examination showed features typical of a neuroendocrine tumour. Immuno-histochemical examination of the resected tumour was positive for neuron specific enolase, chromogranin, insulin, C-peptide, gastrin and somatostatin. Staining was negative for pancreatic polypeptide and VIP.

He remains asymptomatic at 18 months, and CT scan has shown no evidence of recurrent disease.

### DISCUSSION

The clinical manifestation of two hormone syndromes in pancreatic endocrine neoplasia has only been reported with malignant tumours. In a five-year analysis of the results from a supraregional assay service in the U.K., Wynick and colleagues reported that 24 of 353 (6.8%) patients with functioning malignant tumours developed a second hormone syndrome at a median interval of 19 months after initial diagnosis [1]. The most common presenting tumours in this group were glucagonomas, followed by gastrinomas. The development of a second peptide syndrome was associated with marked clinical deterioration and death in over 90% of these patients.

Both our patients showed features of two clinical syndromes. The first patient had gallstones and mild diabetes, typical features of the somatostatinoma syndrome. He did not exhibit diarrhoea, the other typical finding in this syndrome. He subsequently developed severe hypercalcaemia due to secretion of PTHRP, a recently described peptide that mimics the physiological action of parathyroid hormone [2]. PTHRP expression has been demonstrated in both normal and neoplastic islet cell tissue by Asa and colleagues [illegible] In our second patient, the intractable ulcer diathesis and subsequent neuroglycopenic episodes were clear consequences of first gastrin and then insulin hypersecretion. Resection of the pancreatic tumours was associated with decreases in the serum levels of the peptides responsible for the clinical syndromes in both patients. Immunoreactivity for the relevant peptides was confirmed in the excised tumours, with the exception of PTHRP for which immunohistochemical examination was not performed. Concomitant secretion of insulin and gastrin has previously been reported in a small number of cases [4-6]. In an extensive review of the Zollinger-Ellison tumour registry, 2% of patients also had insulin-secreting tumours [7]. Production of PTHRP in pancreatic neuroendocrine tumours in sufficient quantity to cause symptomatic hypercalcaemia has been reported in a few patients [8,9], including one patient in whom concomitant secretion of somatostatin and PTHRP was documented [10].

Secretion of more than one peptide may occur as a result of cellular dysdifferentiation. Small numbers of proliferative cells, uncommitted to a particular differentiation pathway, are present in normal islet tissue and are increased in number in regenerating tissue. If these cells undergo malignant transformation, they may synthesize more than one biocative peptide in sufficient amounts to cause clinical syndromes of peptide excess.

Serum peptide levels should be regularly checked in patients who are being treated for malignant pancreatic neuroendocrine tumours to detect development of a second hormone syndrome. Recent improvements in survival of these patients because of advances in chemotherapy and radiological and surgical interventional techniques may make this a commoner clinical scenario.

### REFERENCES

1. Wynick D, Williams S.J, Bloom S.R. (1988) Symptomatic secondary hormone syndromes in patients with established malignant pancreatic endocrine tumours. *NEJM*, **319**: 605–7.
2. Suva L.J, Winslow G.A, Wettenhall R.E.H, et al. (1987) A parathyroid hormone-related protein implicated in malignant hypercalcemia. *Science*, **237**: 893–6.
3. Asa S.L, Henderson J, Goltzman D, Drucker D.J. (1990) Parathyroid hormone-like peptide in normal and neoplastic human endocrine tissues. *J Clin Endocrinol Metab*, **71**: 1112–1118.
4. Schreiber W. (1963) Insulin producing Zollinger-Ellison tumour. *Surgery*, **55**: 448.
5. Peurifoy J.T, Gomez L.G, Thompson J.C. (1979) Separate pancreatic gastrin cell and beta-cell adenomas. *Arch Surg*, **114**: 956–8.
6. Bar M, Burke M, Isakov A, Almog C. (1990) Insulinoma after streptozotocin therapy for metastatic gastrinoma: natural history or iatrogenic complication. *J Clin Gastroenterol*, **12**: 579–580.
7. Wilson S.D. (1973) Ulcerogenic tumours of the pancreasL the Zollinger-Ellison syndrome. In: Carey LC (ed): The Pancreas. St Louis, CV Mosby Co, pp 295–318.
8. Heath D.A, Senior P.V, Varley J.M, Beck F. (1990) Parathyroid hormone related protein in tumours associated with hypercalcaemia. *Lancet*, **335**: 66–9.
9. Wynick D, Ratcliffe W.A, Heath D.A, Ball S, Barnard M, Bloom S.R. (1990) Treatment of a malignant pancreatic endocrine tumour secreting parathyroid hormone-related protein. *Br. Med. J.*, **300**L: 1314–5.
10. Williams E.J, Ratcliffe W.A, Stavri G.T, Stamatakis J.D. (1992) Hypercalcaemia secondary to secretion of parathyroid hormone-related protein from a somatostatinoma of the pancreas. *Ann Clin Biochem*, **29**: 334–357.

